# Practical Considerations of Remote Care in Thoracic Aortopathy in India

**DOI:** 10.3390/jcm13113327

**Published:** 2024-06-05

**Authors:** Nimrat Grewal, Mohammed Idhrees, Bashi Velayudhan, Robert J. M. Klautz, Simran Grewal

**Affiliations:** 1 Department of Cardiothoracic Surgery, Amsterdam University Medical Center, 1105AZ Amsterdam, The Netherlands; r.j.m.klautz@lumc.nl; 2 Department of Cardiothoracic Surgery, Leiden University Medical Center, 2333 Leiden, The Netherlands; 3 Department of Anatomy and Embryology, Leiden University Medical Center, 2333 Leiden, The Netherlands; 4 Institute of Cardiac and Aortic Disorders (ICAD), SRM Institutes for Medical Science (SIMS Hospital), Chennai 600083, India; a.m.idhrees@gmail.com (M.I.); bashivv@hotmail.com (B.V.); 5 Department of Orthopaedic Surgery, Amsterdam University Medical Center, 1105 Amsterdam, The Netherlands; s.k.grewal@olvg.nl

**Keywords:** thoracic aortopathy, remote care, telemedicine, digital health, patient engagement, technology integration, lifestyle

## Abstract

**Background:** Thoracic aortopathy includes conditions like aortic aneurysms and dissections, posing significant management challenges. In India, care delivery is complicated by geographic vastness, financial constraints, and healthcare resource disparities. Telemedicine and digital health technologies offer promising solutions. **Methods:** A comprehensive review of literature and clinical experiences was conducted to explore the implementation of remote care strategies for thoracic aortopathy in India. The review included studies from 2000 to 2023 and insights from cardiothoracic specialists. **Results:** Remote care benefits include improved access to specialized expertise, enhanced patient engagement, and optimized resource utilization. Telemedicine enables consultations without travel, and remote monitoring facilitates early intervention. However, challenges like technology integration, digital literacy, patient engagement, privacy concerns, and regulatory compliance need addressing. **Discussion:** Telemedicine offers significant advantages but requires overcoming challenges to ensure effective, secure care. Careful planning for technology integration, patient education, robust privacy measures, and supportive regulatory policies are essential. Addressing these issues can bridge the healthcare access gap and improve outcomes in India’s diverse landscape.

## 1. Introduction

Thoracic aortopathy refers to disorders affecting the thoracic aorta, such as aortic aneurysms and dissections. Managing these conditions requires close monitoring, timely intervention, and multidisciplinary care to prevent life-threatening complications [1]. However, delivering optimal care is challenging in remote or underserved areas with limited access to specialized healthcare services.

Healthcare density refers to the availability and distribution of healthcare resources relative to the population. India, with over 1.43 billion inhabitants, has diverse healthcare needs and challenges. Factors such as geography, socioeconomic status, government policies, and healthcare infrastructure development influence healthcare density in India [2]. Despite progress in expanding access to healthcare services, disparities in healthcare density persist, necessitating ongoing investment in infrastructure, workforce development, and healthcare delivery models.

The emergence of telemedicine and digital health technologies offers new opportunities to address these challenges by enabling remote care delivery, consultation, and monitoring [3]. Telemedicine is defined by the World Health Organization as “the delivery of healthcare services, where distance is a critical factor, by all healthcare professionals using information and communication technologies for the exchange of valid information for diagnosis, treatment, and prevention of disease and injuries, research and evaluation, and for the continuing education of healthcare providers, all in the interests of advancing the health of individuals and their communities” [4].

**Figure 1:**
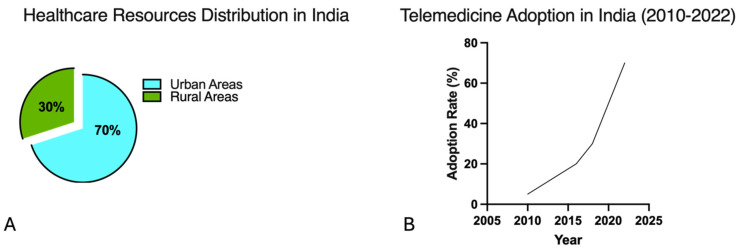
**Healthcare resource distribution and telemedicine adoption in India:** (**A**) shows that approximately 70% of healthcare resources are concentrated in urban areas, while only 30% are available in rural areas. This imbalance highlights the challenges faced in providing equitable healthcare access across the country [8]. The telemedicine market in India reached a valuation of USD 1.10 billion in 2022 and is projected to grow at a compound annual growth rate (CAGR) of 21.2%, reaching USD 5.15 billion by 2030. This growth has been significantly influenced by the COVID-19 pandemic and the subsequent increase in digital health initiatives, such as the National Health Digital Mission, as shown in (**B**) [8,9].

India stands to benefit significantly from telemedicine due to several factors:

1. Geographical Challenges: India’s vast and diverse geography includes many remote areas with limited healthcare access. In India, there is a significant disparity in healthcare resources between urban and rural areas (Figure 1A). Telemedicine can bridge these gaps by offering virtual consultations and medical services, reducing the need for long-distance travel, and improving healthcare accessibility [5].
2. Financial Constraints: Many Indians face financial barriers to quality healthcare. Telemedicine provides a cost-effective alternative to in-person consultations, eliminating travel expenses and hospital visits and making healthcare more affordable and accessible from home [6].
3. Healthcare Density: India faces challenges in healthcare density, especially in rural areas, with around 9 doctors per 10,000 inhabitants. Telemedicine optimizes existing healthcare resources by connecting patients remotely with healthcare providers, extending services to underserved populations, and easing the burden on urban healthcare facilities [2].
4. Improved Health Outcomes: Telemedicine facilitates early diagnosis, timely intervention, and continuity of care, improving health outcomes by preventing complications, reducing hospitalizations, and enhancing patient well-being, which is crucial in managing India’s significant burden of non-communicable diseases [7].
5. Public Health Benefits: Telemedicine supports public health initiatives like disease surveillance, outbreak management, and health education. Digital technologies enable healthcare authorities to monitor trends, disseminate information, and deliver preventive care efficiently, aiding disease prevention and population health management.

India’s telemedicine market, valued at USD 1.10 billion in 2022, is projected to reach USD 5.15 billion by 2030, with a compound annual growth rate (CAGR) of 21.2% (Figure 1B) [8]. The Indian government’s Telemedicine Practice Guidelines, issued in March 2020, allow registered medical practitioners to provide remote healthcare consultations, ensuring patient safety, confidentiality, and quality of care. Various telemedicine platforms and mobile applications have emerged, offering services such as online consultations, e-prescriptions, and medical advice, particularly benefiting underserved areas.

The COVID-19 pandemic has further accelerated the adoption of telemedicine in India, with many healthcare providers and patients relying on remote consultations to minimize virus transmission risks [10]. Telemedicine has proven crucial in maintaining continuity of care during lockdowns and restrictions, underscoring its importance in public health emergencies [11].

This paper aims to provide a mini-review of the practical considerations and challenges associated with implementing remote care strategies in the management of thoracic aortopathy in India by evaluating existing literature and drawing upon clinical experiences. Additionally, we discuss the potential benefits, limitations, and best practices for incorporating remote care into the management of patients with thoracic aortopathy.

## 2. Methods

To explore the practical considerations and challenges of remote care in thoracic aortopathy, we conducted a comprehensive review of existing literature and analyzed clinical experiences. The literature review focused on telemedicine, digital health technologies, and their applications in managing thoracic aortopathy. We included studies published between 2000 and 2023, identified through databases such as PubMed, Google Scholar, and the Cochrane Library.

The inclusion criteria for the studies were:

- Studies discussing the implementation and outcomes of telemedicine in thoracic aortopathy.
- Articles on digital health technologies facilitating remote care for thoracic aortopathy.
- Research focusing on the Indian healthcare context.

We excluded studies that did not focus on thoracic aortopathy or remote care strategies. Data extraction involved identifying key themes, challenges, benefits, and best practices related to remote care in thoracic aortopathy.

Additionally, clinical experiences were gathered from healthcare professionals specializing in cardiothoracic surgery and digital health to provide practical insights into the implementation of remote care strategies.

## 3. Results

### 3.1. Long-Term Management of Thoracic Aortopathy

Histopathological analysis of conditions associated with thoracic aortopathy has shown structural abnormalities of the vascular wall extending over the entire length of the aorta, irrespective of the underlying cause [12,13]. The long-term management of thoracic aortopathy aims to prevent complications, preserve aortic function, and optimize patient health [14,15]. Key aspects include:

1. **Regular monitoring and surveillance:** Lifelong monitoring using echocardiography, CT angiography, or MRI to assess disease progression and detect complications like dissection or rupture. Patients with thoracic aortopathy require continuous monitoring to detect any changes in aortic size or morphology and identify complications such as dissection, rupture, or aneurysm formation. Imaging studies such as echocardiography, computed tomography (CT) angiography, or magnetic resonance imaging (MRI) are typically performed at regular intervals as determined by the patient’s specific condition and risk factors [1].
2. **Blood pressure management:** Strict control of blood pressure is essential for preventing further dilation of the aorta and reducing the risk of aortic dissection or rupture. Patients are often prescribed antihypertensive medications to maintain blood pressure within a target range, and lifestyle modifications such as dietary changes, regular exercise, and stress reduction techniques may also be recommended [16].
3. **Medication management:** Depending on the underlying cause of thoracic aortopathy and the presence of associated conditions, patients may require long-term medication therapy. This may include medications to lower blood pressure, such as beta-blockers or angiotensin-converting enzyme (ACE) inhibitors, as well as medications to reduce the risk of blood clots or manage other cardiovascular risk factors.
4. **Genetic counseling and screening:** Patients with hereditary forms of thoracic aortopathy, such as Marfan syndrome, Loeys-Dietz syndrome, or familial thoracic aortic aneurysm and dissection (FTAAD), may benefit from genetic counseling and screening for family members. Identifying individuals at risk allows for early detection, surveillance, and intervention to prevent or delay the onset of aortic complications [17].
5. **Lifestyle modifications:** Encouraging healthy lifestyle habits is important for overall cardiovascular health and can help reduce the progression of thoracic aortopathy. Patients should be advised to maintain a balanced diet low in sodium and saturated fats, engage in regular physical activity, achieve and maintain a healthy weight, avoid tobacco products, and limit alcohol consumption.
6. **Avoidance of high-impact activities:** Patients with thoracic aortopathy are typically advised to avoid activities that may increase the risk of aortic dissection or rupture, such as heavy lifting, strenuous exercise, or contact sports. Low-impact activities such as walking, swimming, or cycling may be recommended instead [18].
7. **Surgical intervention:** In certain cases, surgical intervention may be necessary to repair or replace the diseased portion of the aorta, particularly if there is evidence of significant dilation, dissection, or impending rupture. Surgical options may include open surgical repair or minimally invasive endovascular procedures, depending on the patient’s specific anatomy and comorbidities.
8. **Psychosocial support:** Living with a chronic condition such as thoracic aortopathy can be challenging, both physically and emotionally. Patients may benefit from access to psychosocial support services, such as counseling, support groups, or online resources, to help cope with the psychological impact of the disease and improve their overall quality of life [19].

### 3.2. Benefits of Remote Care in Thoracic Aortopathy [3,20]:

**Improved access to specialized care:** Telemedicine allows patients in remote or underserved areas to access specialized expertise and consultation from thoracic aorta specialists without the need for travel.

**Enhanced monitoring and follow-up:** Remote monitoring tools enable regular surveillance of aortic dimensions and other relevant parameters, facilitating early detection of changes and timely intervention.

**Increased patient engagement:** Digital health platforms can empower patients to actively participate in their care through educational resources, self-monitoring tools, and remote communication with healthcare providers.

**Optimized resource utilization:** Remote consultations and monitoring reduce the need for in-person visits, making healthcare delivery more efficient and cost-effective.

### 3.3. Challenges and Considerations of Remote Care in Thoracic Aortopathy [3,20,21]:

**Technology integration:** Ensuring seamless integration of telemedicine platforms with existing health record systems and imaging modalities.

**Patient engagement and education:** Digital literacy and effective patient engagement and education are essential for the success of remote care initiatives. Strategies for promoting patient adherence, understanding, and empowerment must be developed and tailored to the specific needs of patients with thoracic aortopathy.

**Privacy and security:** Implementing robust security measures and complying with privacy regulations to safeguard patient data.

**Regulatory and reimbursement Issues:** Implementing robust security measures and complying with privacy regulations to safeguard patient data.

## 4. Discussion

This review offers a detailed examination of literature and clinical experiences to investigate the implementation of remote care strategies for thoracic aortopathy in India. The management of thoracic aortopathy using telemedicine is a relatively unexplored area. Our objective is to provide an overview of the long-term management of thoracic aortopathy, highlight the benefits and challenges of remote care in India, and suggest possible future directions for its implementation.

The implementation of remote care strategies in thoracic aortopathy offers numerous benefits, including improved access to specialized care, enhanced monitoring and follow-up, increased patient engagement, and optimized resource utilization. Telemedicine allows patients in remote or underserved areas to access specialized expertise and consultation from thoracic aorta specialists without the need for travel [8,9,22]. Remote monitoring tools enable regular surveillance of aortic dimensions and other relevant parameters, facilitating early detection of changes and timely intervention.

Despite the potential benefits, several challenges and practical considerations must be addressed when implementing remote care strategies in thoracic aortopathy. Technology integration, internet connectivity, and the integration of telemedicine platforms with existing electronic health record systems and imaging modalities require careful planning and coordination to ensure seamless workflow and data interoperability. Additionally, digital literacy and effective patient engagement and education are essential for the success of remote care initiatives. Strategies for promoting patient adherence, understanding, and empowerment must be developed and tailored to the specific needs of patients with thoracic aortopathy.

Privacy and security concerns are paramount in remote care delivery [23]. Compliance with privacy regulations (e.g., HIPAA) and safeguarding patient data require robust security measures and encryption protocols to protect patient privacy and confidentiality. Furthermore, regulatory requirements and reimbursement policies vary across jurisdictions and healthcare systems. Clear guidelines and reimbursement mechanisms for remote care services need to be established to support widespread adoption and sustainability.

Our study adds to the existing literature by highlighting the practical considerations and challenges of implementing remote care strategies in thoracic aortopathy, particularly in the context of India. By addressing these challenges and adopting best practices, remote care has the potential to revolutionize the delivery of care in thoracic aortopathy and improve patient outcomes.

## 5. Limitations

This study is based on a review of existing literature and clinical experiences, which may not capture all potential challenges and considerations. Future research should focus on empirical studies to validate the findings and explore additional factors influencing the implementation of remote care in thoracic aortopathy.

## 6. Future Directions

Continued innovation in telemedicine technologies, remote monitoring devices, and digital health solutions is needed to address evolving clinical needs and improve the delivery of remote care in thoracic aortopathy. Interdisciplinary collaboration, continuous quality improvement, and patient-centered care principles should be prioritized to optimize clinical outcomes and patient satisfaction.

## 7. Conclusions

**Figure 2:**
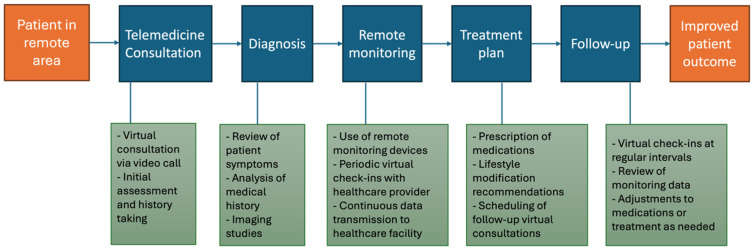
**Flowchart for the remote care process in thoracic aortopathy:** The process begins with a patient located in a remote or underserved area who needs specialized care for thoracic aortopathy. The patient connects with a healthcare provider via a telemedicine platform for an initial consultation (Telemedicine Consultation). Based on the consultation, the healthcare provider makes a preliminary diagnosis. The patient is enrolled in a remote monitoring program to regularly track aortic dimensions and other relevant parameters (Remote Monitoring). A personalized treatment plan is developed based on the diagnosis and monitoring data. Regular follow-up consultations are conducted to review the patient’s progress and make necessary adjustments to the treatment plan. The process aims to achieve improved patient outcomes through continuous monitoring, timely interventions, and personalized care.

Telemedicine offers a viable solution to address the challenges posed by its geographical vastness, financial constraints, and healthcare density [2,5,6,7]. In recent years, remote care has made significant strides in India, driven by regulatory reforms, technological advancements, and changing healthcare needs [8]. While there are still challenges to address, telemedicine holds immense potential to revolutionize healthcare delivery, improve patient outcomes, and enhance healthcare access for patients with thoracic aortopathy by overcoming barriers to access, enhancing patient engagement, and optimizing resource utilization [1,3,12,13,14,15,16,17,18,19,20,21]. By harnessing the power of telecommunication technologies, India can extend the reach of healthcare services, improve health outcomes, and promote equitable access to quality healthcare for all its citizens, regardless of their location or socioeconomic status [3,20,22]. Successful implementation of remote care strategies requires careful consideration of practical factors, including technology integration, patient engagement, privacy concerns, and regulatory requirements. By addressing these challenges and adopting best practices, remote care has the potential to revolutionize the delivery of care in thoracic aortopathy and improve patient outcomes. We propose a flowchart for the remote care process in thoracic aortopathy in Figure 2.

## Data Availability

No new data were created or analyzed in this study. Data sharing is not applicable to this article.
